# Abnormal composition of gut microbiota is associated with resilience versus susceptibility to inescapable electric stress

**DOI:** 10.1038/s41398-019-0571-x

**Published:** 2019-09-17

**Authors:** Kai Zhang, Yuko Fujita, Lijia Chang, Youge Qu, Yaoyu Pu, Siming Wang, Yukihiko Shirayama, Kenji Hashimoto

**Affiliations:** 1 Division of Clinical Neuroscience, Chiba University Center for Forensic Mental Health, Chiba, 260-8670 Japan; 2 Department of Psychiatry, Chaohu Hospital of Anhui Medical University, 238000 Hefei, China; 3 Department of Psychiatry, Teikyo University Chiba Medical Center, Ichihara, Chiba, 299-0111 Japan

**Keywords:** Pharmacodynamics, Depression

## Abstract

Increasing evidence indicates that abnormalities in the composition of gut microbiota might play a role in stress-related disorders. In the learned helplessness (LH) paradigm, ~60–70% rats are susceptible to LH in the face of inescapable electric stress. The role of gut microbiota in susceptibility in the LH paradigm is unknown. In this study, male rats were exposed to inescapable electric stress under the LH paradigm. The compositions of gut microbiota and short-chain fatty acids were assessed in fecal samples from control rats, non-LH (resilient) rats, and LH (susceptible) rats. Members of the order *Lactobacillales* were present at significantly higher levels in the susceptible rats than in control and resilient rats. At the family level, the number of *Lactobacillaceae* in the susceptible rats was significantly higher than in control and resilient rats. At the genus level, the numbers of *Lactobacillus*, *Clostridium* cluster III, and *Anaerofustis* in susceptible rats were significantly higher than in control and resilient rats. Levels of acetic acid and propionic acid in the feces of susceptible rats were lower than in those of control and resilient rats; however, the levels of lactic acid in the susceptible rats were higher than those of control and resilient rats. There was a positive correlation between lactic acid and *Lactobacillus* levels among these three groups. These findings suggest that abnormal composition of the gut microbiota, including organisms such as *Lactobacillus*, contributes to susceptibility versus resilience to LH in rats subjected to inescapable electric foot shock. Therefore, it appears likely that brain–gut axis plays a role in stress susceptibility in the LH paradigm.

## Introduction

Resilience is adaptation in the face of stress and adversity. It is well known that humans display wide variability in their responses to stress. Increasing amounts of evidence show that resilience might be mediated by adaptive changes in several neural circuits, including numerous molecular and cellular pathways^1–11^. An understanding of the molecular and cellular mechanisms underlying resilience will facilitate the discovery of new therapeutic drugs for stress-related psychiatric disorders, but the detailed mechanisms underlying resilience and susceptibility remain unclear.

The brain–gut–microbiome axis is a complex, bidirectional signaling system between the brain and the gut microbiota^12–16^. Accumulating studies suggest that an abnormal composition of the gut microbiota contributes to the pathophysiology of depression^17–21^ and the antidepressant effects of certain potential compounds^22–29^. Previously, we reported that the presence of *Bifidobacterium* in the gut microbiome confers stress resilience in a chronic social defeat stress (CSDS) model^30^. It has been shown that ~30–40% of rats are resilient to inescapable electric stress in the learned helplessness (LH) paradigm^31–34^; however, the role of gut microbiota in the production of this resilience has not yet been investigated. Microbes in the gut can produce short-chain fatty acids. The presence and abundance of such acids could possibly be used as an indicator of the types of bacteria present in the gut. However, it is also currently unknown how the altered composition of the gut microbiota affects the concentration of short-chain fatty acids in fecal samples.

The purpose of this study was to investigate the role of gut microbiota on stress resilience using a rat LH paradigm. First, we investigated whether the composition of the gut microbiota was altered in fecal samples from LH (susceptible) and non-LH (resilient) rats compared with control rats. Then we examined whether the levels of short-chain fatty acids—acetic acid, propionic acid, butyric acid, lactic acid, and succinic acid—in the fecal samples from susceptible and resilient rats were altered compared with control rats, since these short-chain fatty acids can produced by the gut microbiota^23^.

## Materials and methods

### Animals

Male Sprague-Dawley rats (*n* = 25, 200–230 g; 7 weeks, Charles River Japan, Co., Tokyo, Japan) were used. The animals were housed under a 12-h light/dark cycle with ad libitum access to food and water. The experimental procedures were approved by the Chiba University Institutional Animal Care and Use Committee (Permission number: 31-341).

### Stress paradigm (LH model) and collection of fecal sample

The LH paradigm was performed as previously reported^6–8,11,31–34^. Animals were initially exposed to uncontrollable stress to create LH rats. When the rats were later placed in a situation in which the shock is controllable; that is, the animal could escape it, an animal exhibiting LH not only fails to acquire an escape response but also often makes no effort to escape the shock at all.

**Fig. 1:**
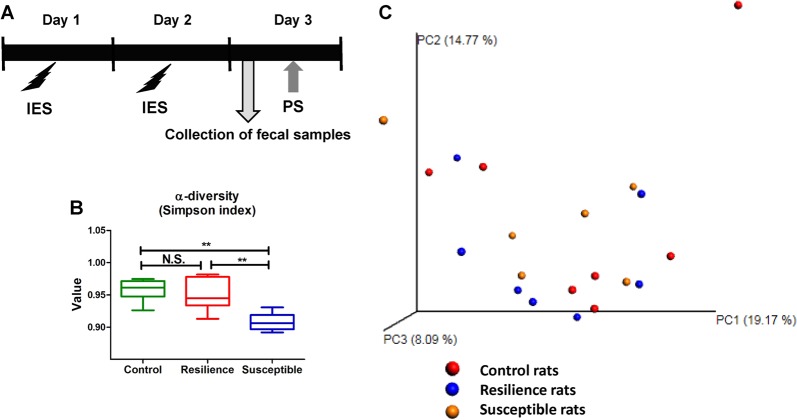
Experimental schedule of LH paradigm and profiles of gut microbiota. **a** Schedule of LH paradigm and collection of fecal samples. Rats received inescapable electric shock (IES) treatments on 2 days (days 1 and 2). On day 3, fecal samples from rats were collected. Subsequently, rats passed a post-shock test (PS), and were designated as resilient rats and susceptible rats. **b** Simpson index (an α-diversity indicator: one-way ANOVA: *F*_2,17_ = 11.531, *P* = 0.001) among the three groups. α-Diversity data are shown as mean ± S.E.M. (*n* = 6 or 7). **P < 0.01. N.S.: not significant. **c** Principal coordinates analysis (PCA)

We used the Gemini Avoidance System (San Diego Instruments, San Diego, CA) for LH paradigm. This apparatus has two compartments by a retractable door. On day 1 and day 2, rats were subjected to 30 inescapable electric foot shocks (0.65 mA, 30 s duration, at random intervals averaging 18–42 s). On day 3, a post-shock test using a two-way conditioned avoidance test was performed to determine whether the rats would exhibit the predicted escape deficits (Fig. 1a). This session consisted of 30 trials, in which electric foot shocks (0.65 mA, 6 s duration, at random intervals with a mean of 30 s) were preceded by a 3-s conditioned stimulus tone that remained on until the shock was terminated. The numbers of escape failures and the latency to escape in each of the 30 trials were counted. Animals with more than 25 escape failures in the 30 trials were regarded as having met the criterion for LH rats (susceptible). Animals with fewer than 24 failures were defined as non-LH rats (resilient)^31–34^. Fresh fecal samples were collected in a blind manner before post-shock stress on day 4, and stored at −80 °C until use (Fig. 1A).

### 16S rDNA analysis

DNA extraction from fecal samples and the 16S rDNA analysis were performed at the TechnoSuruga Laboratory, Co., Ltd. (Shizuoka, Japan), as reported previously^35^. Briefly, the samples were suspended in a buffer containing 4 M guanidium thiocyanate, 100 mM Tris-HCl (pH 9.0) and 40 mM EDTA and broken up in the presence of zirconia beads using the FastPrep-24 5G homogenizer (MP Biomedicals, Irvine, CA). Then, DNA was extracted using GENE PREP STAR PI-480 (KURABO, Japan). The final concentration (10 ng/μL) of the DNA sample was used. Briefly, the V3-V4 hypervariable regions of the 16S rRNA were amplified from microbial genomic DNA using PCR with the bacterial universal primers (341F/R806)^35^ and the dual-index method^36^. For bioinformatics analysis, the overlapping paired-end reads were merged using the fastq-join program with default settings^37^. The reads were processed for quality and chimera filtering as follows. Only reads with quality value scores of 20 for >99% of the sequence were extracted, and chimeric sequences were removed using the program usearch6.1^38^. Non-chimeric reads were submitted for 16S rDNA-based taxonomic analysis using the Ribosomal Database Project (RDP) Multiclassifier tool^39^. Reads obtained in the Multi-FASTA format were assigned to genus or phylum levels with an 80% confidence threshold. Principal component analysis (PCA) was performed using Metagenome@KIN software (World Fusion Co., Ltd., Tokyo, Japan) based on data obtained from the bacterial family using the RDP taxonomic analysis software.

### Measurement of fecal short-chain fatty acids

Measurement of short-chain fatty acids—acetic acid, propionic acid, butyric acid, lactic acid, and succinic acid—in fecal samples was performed at the TechnoSuruga Laboratory, Co., Ltd. (Shizuoka, Japan). For the determination of these short-chain fatty acids, feces were suspended in distilled water, heated at 85 °C for 15 min to inactivate viruses, and then centrifuged according to previously reported methods^40,41^. The concentrations of these short-chain fatty acids in feces were measured using a high-performance liquid chromatography organic acid analysis system with a Prominence CDD-10A conductivity detector (Shimadzu, Kyoto, Japan), two tandemly arranged Shim-pack SCR-102(H) columns [300 mm × 8 mm inner diameter (ID)], and a Shim-pack SCR-102(H) guard column (50 mm × 6 mm ID)^40,41^. The HPLC calibration curves for the measurement of the short-chain fatty acids were created using prepared standard solutions.

### Statistical analysis

The data are presented as the mean ± standard error of the mean (S.E.M.). Analysis was performed using the PASW Statistics 20 software (now SPSS statistics; SPSS, Tokyo, Japan). Comparisons between groups were performed using one-way analysis of variance, followed by post hoc Fisher’s Least Significant Difference test. A *P* value < 0.05 was considered statistically significant.

## Results

### Composition of gut microbiota in control, resilient, and susceptible rats

We used 16S rDNA gene sequencing to determine differences in the gut microbiota composition among the three groups of rats. α-diversity refers to the diversity of bacteria or species within a community or habitat. The susceptible rats showed a significant decrease in the α-diversity value compared with control rats or resilient rats (Fig. 1b). In the three-dimensional PCoA data, the measurements from susceptible rats were well separated from those of control rats and resilient rats. Four measurements from the susceptible group were close to those of the sham group, whereas the other three measurements were close to those of the resilient group (Fig. 1c).

**Fig. 2:**
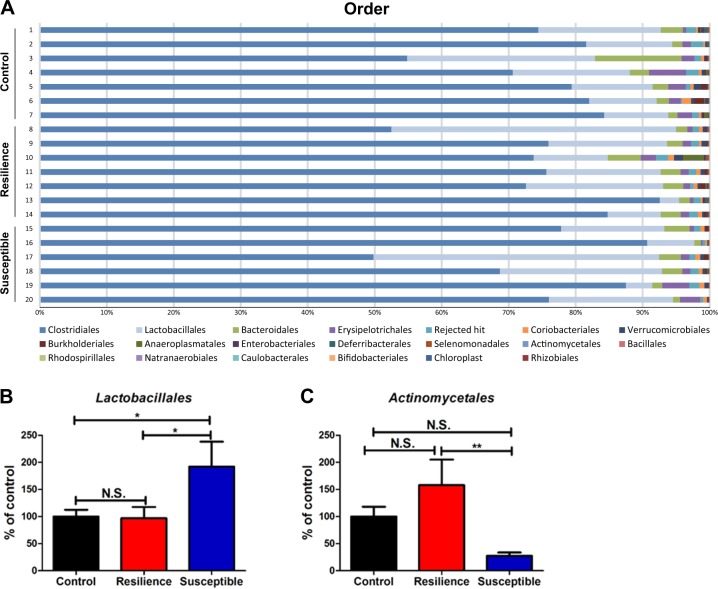
Changes in the composition of gut microbiota at the order level. **a** The order levels of gut microbiota among the three groups. **b** *Lactobacillales* (one-way ANOVA: *F*_2,17_ = 4.021, *P* = 0.044) among the three groups. **c** *Actinomycetales* (one-way ANOVA: *F*_2,17_ = 4.000, *P* = 0.038) among the three groups. The data are shown as mean ± S.E.M. (*n* = 6 or 7). *P < 0.05, **P < 0.01. N.S.: not significant

*Firmicutes* were the most dominant phylum, comprising >85% of the total sequences. There were no significant differences in the levels of this phylum among the three groups. The order levels of gut bacterium in control rats, resilient rats, and susceptible rats were identified (Fig. 2a). *Clostridiales* and *Lactobacillales* were the most dominant orders, with >80% of total sequences. The number of *Lactobacillales* was significantly increased in the susceptible rats compared with that in control and resilient rats (Fig. 2b). In contrast, the number of *Lactobacillales* in the resilient rats was similar to that in the control rats (Fig. 2b). The number of *Actinomycetales* in the susceptible rats was significantly lower than that in the resilient rats (Fig. 2c). Although the number of *Actinomycetales* in the resilient rats was higher than that in the control rats, the difference did not reach statistical significance.

**Fig. 3:**
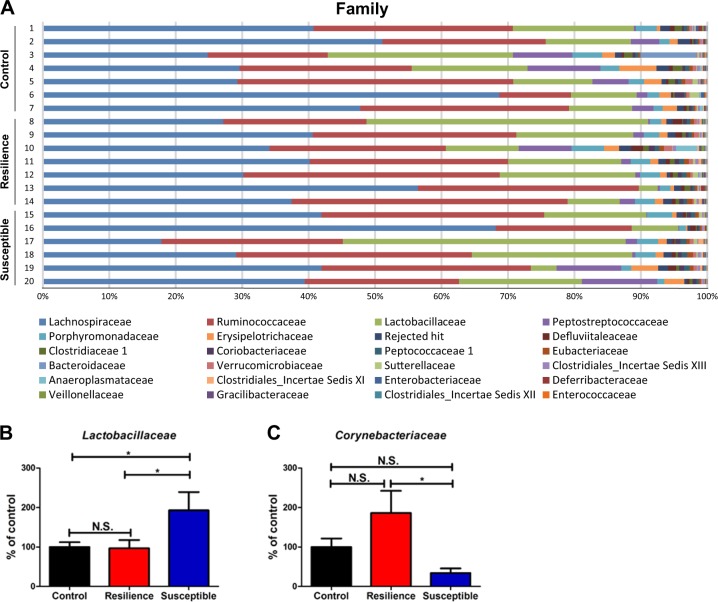
Changes in the composition of gut microbiota at the family level. **a** The family levels of gut microbiota among the three groups. **b** *Lactobacillaceae* (one-way ANOVA: *F*_2,17_ = 4.039, *P* = 0.043) among the three groups. **c** *Corynebacteriaceae* (one-way ANOVA: *F*_2,17_ = 4.159, *P* = 0.034) among the three groups. The data are shown as mean ± S.E.M. (*n* = 6 or 7). *P < 0.05, **P < 0.01. N.S.: not significant

The families of the gut bacteria in control, resilient, and susceptible rats are shown (Fig. 3a). *Lactobacillaceae* were significantly more highly represented in the susceptible rats than in the control and resilient rats, although the number of *Lactobacillaceae* in the resilient rats was similar to that in the control rats (Fig. 3b). In contrast, the number of *Corynebacteriaceae* was significantly lower in the susceptible rats than that in the resilient rats, although these two did not significantly differ from the control rats (Fig. 3c).

**Fig. 4:**
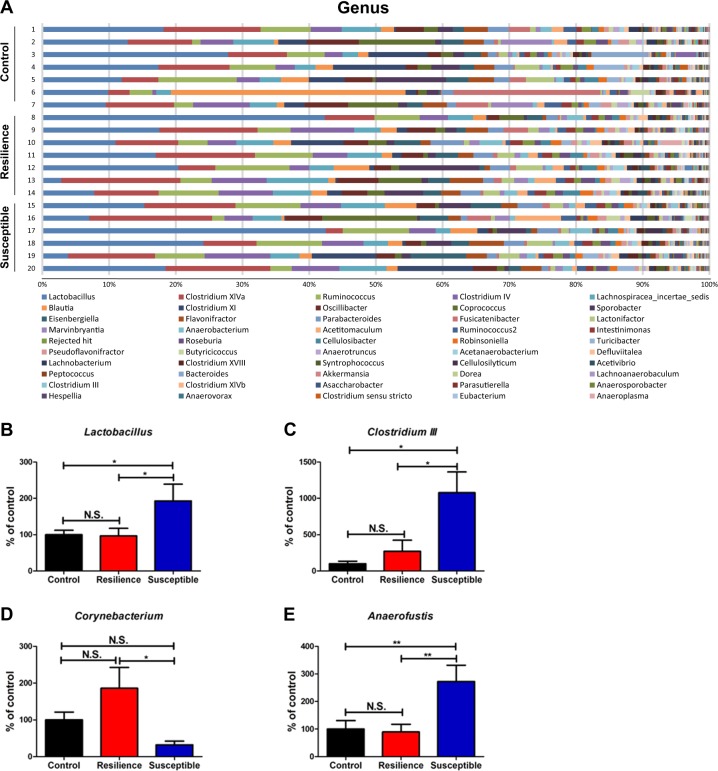
Changes in the composition of gut microbiota at the genus level. **a** The genus levels of gut microbiota among the three groups. **b** *Lactobacillus* (one-way ANOVA: *F*_2,17_ = 4.030, *P* = 0.043) among the three groups. **c** *Clostridium III* (one-way ANOVA: *F*_2,17_ = 5.625, *P* = 0.021) among the three groups. **d** *Corynebacterium* (one-way ANOVA: *F*_2,17_ = 4.283, *P* = 0.031) among the three groups. **c** *Anaerofustis* (one-way ANOVA: *F*_2,17 _= 6.608, *P* = 0.008) among the three groups. The data are shown as mean ± S.E.M. (*n* = 6 or 7). *P < 0.05, **P < 0.01. N.S.: not significant

The genera of gut bacteria in the control, resilient, and susceptible rats are shown (Fig. 4a). *Lactobacillus*, *Clostridium* cluster III, and *Anaerofustis* numbers were significantly higher in the susceptible rats than in the control and resilient rats (Fig. 4b, c, e). *Corynebacterium* numbers were significantly lower in the susceptible rats than in the resilient rats, although these two groups were not significantly altered compared with the control rats (Fig. 4d).

### Measurement of short-chain fatty acids in fecal samples

**Fig. 5:**
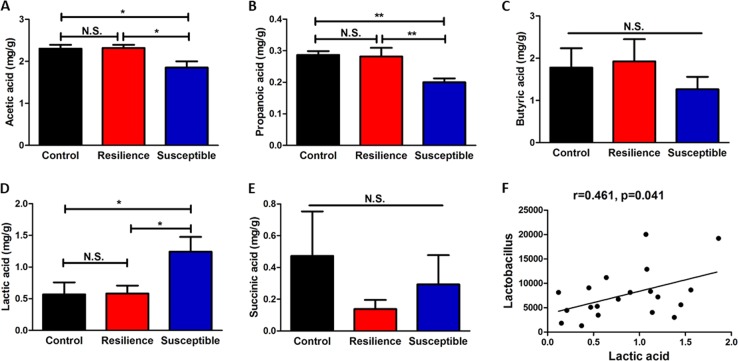
Levels of short-chain fatty acids in fecal samples and correlation with microbiota. **a** Acetic acid (one-way ANOVA: *F*_2,17_ = 5.898, *P* = 0.016) among the three groups. **b** Propionic acid (one-way ANOVA: *F*_2,17_ = 8.175, *P* = 0.004) among the three groups. **c** Butyric acid (one-way ANOVA: *F*_2,17_ = 0.559, *P* = 0.582) among the three groups. **d** Lactic acid (one-way ANOVA: *F*_2,17_ = 4.219, *P* = 0.041) among the three groups. **e** Succinic acid (one-way ANOVA: *F*_2,17_ = 0.763, *P* = 0.488) among the three groups. The data are shown as mean ± S.E.M. (*n* = 6 or 7). *P < 0.05, **P < 0.01. N.S.: not significant. **f** There is a positive correlation (*r* = 0.461, *P* = 0.041) between lactic acid and Lactobacillus in fecal samples

Levels of acetic acid and propionic acid in the susceptible rats were significantly lower than those in control rats and resilient rats, and there were no significant differences between control rats and resilient rats (Fig. 5a, b). There were no changes in butyric acid and succinic acid among the three groups (Fig. 5c, e). In contrast, levels of lactic acid in the susceptible rats were significantly higher than those of control rats and resilient rats, although there were no changes between control rats and resilient rats (Fig. 5d). There was a positive correlation (*r* = 0.461, *P* = 0.041) between lactic acid and *Lactobacillus* levels among three groups (Fig. 5f). There were no correlations between other short-chain fatty acids and the microbiome composition among the three experimental groups.

## Discussion

The major findings of this study are as follows. At the order level, susceptibility to LH in rats exposed to inescapable shock might be associated with an increase of *Lactobacillales* and a decrease of *Actinomycetales* in the host gut. At the family level, stress susceptibility might be associated with an increase in *Lactobacillaceae* and decrease of *Corynebacteriaceae* in the host gut. At the gene level, stress susceptibility might be associated with the increase of *Lactobacillus*, *Clostridium* cluster III and *Anaerofustis*, and the decrease of *Corynebacterium* in the host gut. Levels of acetic acid and propionic acid in the feces from susceptible rats were lower than those in the feces of control and resilient rats, whereas levels of lactic acid in the susceptible rats were higher than those of control and resilient rats. There was a positive correlation between lactic acid and *Lactobacillus* levels among these three groups. These findings suggest that alterations in the composition of these microbiota contribute to susceptibility versus resilience in rats in the LH situation.

In this study, we found an increase in the abundance of members of the order *Lactobacillales* and the family *Lactobacillaceae* in susceptible rats, in comparison with control and resilient rats. It is possible that increased abundance of members of the *Lactobacillales* order and *Lactobacillaceae* family might play a role in susceptibility versus resilience of rats to LH after inescapable electric stress.

At the genus level, susceptibility in rats exposed to inescapable shock might be associated with an increase in *Lactobacillus*, *Clostridium* cluster III, and *Anaerofustis* and a decrease of *Corynebacterium* in the host gut. *Clostridium*, a genus of Gram-positive bacteria, includes several significant human pathogens, such as the causative agent of botulism. High levels of members of the genus *Clostridium* have been reported in patients with major depressive disorder (MDD) compared with controls^42,43^, suggesting that increased levels of *Clostridium* play a role in depression. We reported that susceptible mice after CSDS have higher levels of *Clostridium*, and that the novel antidepressant candidate (*R*)-ketamine attenuated the increased levels of *Clostridium* in susceptible mice^25^. This study shows that the antidepressant effects of (*R*)-ketamine might be partly mediated by the restoration of altered composition of the gut microbiota in the CSDS susceptible mice. Although the role of members of *Clostridium* cluster III in depression is currently unclear, it appears that *Clostridium* cluster III may contribute to susceptibility in rats subjected to inescapable electric stress.

*Anaerofustis* is a strictly anaerobic, Gram-positive, rod-shaped, non-spore-forming bacterial genus of the family *Eubacteriaceae*^44^. In this study, we found decreased levels of *Anaerofustis* in the susceptible rats compared with the control and resilient rats. At present, there have been no reports showing alterations in *Anaerofustis* in patients with MDD, or in rodents with depression-like phenotypes. Therefore, it is unclear how decreased levels of *Anaerofustis* play a role in susceptibility to LH. Further study into the role of *Anaerofustis* in depression is needed.

In this study, we found lower levels of *Corynebacterium* in the susceptible rats compared with control and resilient rats. It has been reported that sub-chronic and chronic exposure to glyphosate-based herbicides decreases the composition of microbiota, such as *Corynebacterium*, resulting in behavioral abnormalities including depression and anxiety^45^. Low levels of *Corynebacterium* were also reported in a chronic variable stress-induced rat model of depression^46^. It appears likely that lowered levels of *Corynebacterium* might play a role in a depression-like phenotype in rodents, although further study into the role of *Corynebacterium* in depression is needed.

Short-chain fatty acids—acetic acid, propionic acid, butyric acid, lactic acid, and succinic acid—are generated as the end products of the degradation and fermentation of indigestible carbohydrates by the gut microbiota^47^. Measurement of these short-chain fatty acids could therefore serve as an indirect method for the analysis of microbiota composition. These organic acids have specific anti-microbial activities^48^. It has been reported that levels of acetic acid and propionic acid in feces from women with depression are lower than those in control subjects, and that there are negative correlations between acetic acid or propionic acid and depression scores^49^. In this study, we found decreased levels of acetic acid and propionic acid in rats susceptible to LH, compared with control and resilient rats, consistent with the results from a recent clinical study^49^. We also found decreased levels of butyric acid in the susceptible rats, although the difference did not reach statistical significance. Three short-chain fatty acids containing C2–C4, acetic acid, propionic acid, and butyric acid, account for over 95% of the pool of short-chain fatty acids^23^. It is likely that decreased levels of acetic acid and propionic acid in feces may be associated with susceptibility to LH, and with depression in patients with MDD.

Elevated levels of lactic acid in the blood, cerebrospinal fluid and brain have been reported in patients with MDD^50–52^. Higher levels of lactic acid in rodents with depression-like behaviors have also been reported^53^. In contrast, peripheral administration of lactic acid produced antidepressant-like effects in different models of depression^54^. In this research we found higher levels of lactic acid in feces from susceptible rats compared with those from control and resilient rats. We found a positive correlation between *Lactobacillus*, microbes which produce lactic acid, and the amounts of lactic acid in fecal samples. It appears likely that the increased levels of lactic acid produced by *Lactobacillus* might contribute to susceptibility to inescapable electric stress, although further study is needed.

The crosstalk between the brain and the gut is predominately influenced by the gut bacteria^55^. Imbalance of gut microbiota has been found to cause abnormalities in the brain–gut axis in several neurological and psychiatric diseases^13,55^. Multiple lines of evidence suggest that an abnormal composition of the gut microbiota contributes to the resilience or susceptibility to LH in rodents after repeated stress^30,56–59^. It is well recognized that gut microbiota plays a role in animal behaviors^14,60–62^, although the precise mechanisms underlying the microbiome-mediated behaviors are currently unknown. For example, it has been reported that the vagus nerve plays a major role in modulating the constitutive communication pathway between the brain and the bacteria in the gut^63,64^. It is likely that altered composition of microbiota might play a key role in the stress-induced disorders although further study is needed.

This research has some limitations. In this study, we did not identify the specific microbiome which can affect susceptibility or resilience in the LH experiments. Therefore, from the present data, we do not know how the specific microbiome can affect behaviors under the LH paradigm. In the future, it will be necessary to identify the specific microbiome, using approaches such as shotgun metagenomics sequencing. It will also be of interest to investigate the way in which specific microbiomes affect behaviors related to LH.

In conclusion, the present study suggests that an altered composition of the gut microbiota, including organisms, such as *Lactobacillus*, *Clostridium* cluster III, *Anaerofustis*, and *Corynebacterium*, contributes to resilience and susceptibility to learned helplessness in rats subjected to inescapable electric foot shock.
